# MolMVC: Enhancing molecular representations for drug-related tasks through multi-view contrastive learning

**DOI:** 10.1093/bioinformatics/btae386

**Published:** 2024-09-04

**Authors:** Zhijian Huang, Ziyu Fan, Siyuan Shen, Min Wu, Lei Deng

**Affiliations:** School of Computer Science and Engineering, Central South University, Changsha 410083, China; School of Computer Science and Engineering, Central South University, Changsha 410083, China; School of Computer Science and Engineering, Central South University, Changsha 410083, China; Institute for Infocomm Research, Agency for Science, Technology and Research (A*STAR), Singapore 138632, Singapore; School of Computer Science and Engineering, Central South University, Changsha 410083, China

## Abstract

**Motivation:**

Effective molecular representation is critical in drug development. The complex nature of molecules demands comprehensive multi-view representations, considering 1D, 2D, and 3D aspects, to capture diverse perspectives. Obtaining representations that encompass these varied structures is crucial for a holistic understanding of molecules in drug-related contexts.

**Results:**

In this study, we introduce an innovative multi-view contrastive learning framework for molecular representation, denoted as MolMVC. Initially, we use a Transformer encoder to capture 1D sequence information and a Graph Transformer to encode the intricate 2D and 3D structural details of molecules. Our approach incorporates a novel attention-guided augmentation scheme, leveraging prior knowledge to create positive samples tailored to different molecular data views. To align multi-view molecular positive samples effectively in latent space, we introduce an adaptive multi-view contrastive loss (AMCLoss). In particular, we calculate AMCLoss at various levels within the model to effectively capture the hierarchical nature of the molecular information. Eventually, we pre-train the encoders via minimizing AMCLoss to obtain the molecular representation, which can be used for various down-stream tasks. In our experiments, we evaluate the performance of our MolMVC on multiple tasks, including molecular property prediction (MPP), drug-target binding affinity (DTA) prediction and cancer drug response (CDR) prediction. The results demonstrate that the molecular representation learned by our MolMVC can enhance the predictive accuracy on these tasks and also reduce the computational costs. Furthermore, we showcase MolMVC’s efficacy in drug repositioning across a spectrum of drug-related applications.

**Availability and implementation:**

The code and pre-trained model are publicly available at https://github.com/Hhhzj-7/MolMVC.

## 1 Introduction

Molecular representation holds a foundational role in drug discovery and the comprehension of molecular behavior (Li *et al.* 2021). A robust molecular representation proves invaluable for various downstream tasks, spanning molecular property prediction and a spectrum of drug-related tasks. However, prevailing approaches for addressing these tasks often necessitate the development of specialized modules to extract molecular information from drugs or rely on conventional molecular representations like fingerprints (Rogers and Hahn 2010). Nevertheless, insights derived solely from task-specific datasets are inherently limited, and traditional fingerprints lack the capacity to encode the intricate topology and geometry details of molecules. Concurrently, the abundance of available molecular data (Kim *et al.* 2021) contributes to a colossal latent space. Hence, a critical research focus lies in devising methodologies to obtain high-quality representations that encapsulate comprehensive and multi-view information of molecules.

The pretraining-finetuning pipeline stands out as a typical approach in transfer learning. This sequential process entails initially using a pre-training scheme to glean informative representations from extensive unlabeled data, followed by a fine-tuning scheme that refines these representations with specific information from limited labeled data. Widely successful in various domains, such as Natural Language Processing and Computer Vision, this methodology is particularly relevant for molecular data given the substantial gap between the sizes of unlabeled and labeled datasets. To attain high-quality molecular representations for augmenting downstream tasks like Molecular Property Prediction (MPP), drug-related tasks, and others, it becomes imperative to comprehensively capture the inherent features of molecules during the pre-training phase. Contrastive learning (CL), a notable paradigm within the realm of self-supervised learning (SSL), is designed to instruct a model in discerning between positive and negative sample pairs (Jaiswal *et al.* 2020). This approach is well-suited for pre-training and has demonstrated promising performance in the domain of molecular representation learning.

Thus far, contrastive learning-based methods for molecular representation learning can be mainly categorized into two strategies. The first strategy involves leveraging diverse data augmentation schemes to construct positive sample pairs. For instance, You *et al.* (2020) introduce a graph contrastive learning framework for generating molecular representations. They propose four general data augmentations tailored for graph-structured data, encompassing node dropping, edge perturbation, attribute masking, and subgraphs. Another notable contribution is from Suresh *et al.* (2021). They present AD-GCL, which incorporates a learnable edge-dropping augmentation, randomly removing edges through a Bernoulli distribution with trainable parameters from a Graph Neural Networks augmenter. In addition, Xia *et al.* (2023) contribute Mole-BERT, a pre-training framework adopting triplet masked contrastive learning. In this approach, atoms are randomly masked with different masking ratios, simulating diverse semantic similarities among molecules. The second strategy involves constructing positive sample pairs by leveraging different data modalities of molecules. While many contrastive learning methods for molecular representation traditionally focus on the 2D topological structure of molecules, it is imperative to recognize, from a bioinformatics perspective, that the efficacy of drugs and molecular properties is intricately linked to the 3D geometry structure of molecules (Liu *et al.* 2021b). For example, Liu *et al.* (2021a) propose GraphMVP which uses contrastive learning on 2D representation and 3D representation to utilize inter-molecule knowledge. Meanwhile, Stärk *et al.* (2022) present 3D Informax, which utilizes the 3D structure of molecules and incorporates molecular conformers within the contrastive learning.

While previous studies have made notable advancements, there is still ample room for further enhancements and refinements. (i) Existing research primarily concentrates on partial formulations of molecules, neglecting a holistic consideration of 1D, 2D, and 3D perspectives. (ii) The majority of augmentation approaches rely on random masking, disregarding the semantic intricacies within molecules. This can lead to the generation of molecular augmented samples with unstable quality, consequently impacting the accuracy of the model’s learned molecular semantics. (iii) Contrastive learning applied to molecular representations of various modalities enables the model to grasp information from different views. Simultaneously, constructing augmented samples for contrastive learning facilitates the model in learning information specific to each view. Developing a contrastive learning method that effectively integrates both aspects proves advantageous in obtaining representations enriched with comprehensive information. (iv) Previous research has overlooked molecular information at different hierarchical levels, resulting in pre-trained models lacking the capacity to discern information at varying scales. For instance, considering the atomic information required for the entire molecule must differ from focusing only on neighboring atoms.

In this study, we propose MolMVC, an innovative multi-view contrastive learning framework that fully focuses on molecular self information, aiming to enhance molecular representation and improve performance on drug-related tasks. Our approach is tailored to accommodate the diverse data patterns associated with different information perspectives inherent in molecular structures. To capture the intrinsic features of molecules comprehensively, we use a Transformer encoder to encode the 1D Explainable Substructure Partition Fingerprint (ESPF) (Huang *et al.* 2019), and a Graph Transformer to encode the 2D topology graph and 3D geometry graph. To facilitate effective learning, we introduce a novel contrastive loss, AMCLoss, specifically designed to bring closer both the formulation representations of the same molecule and their corresponding augmentation representations in the latent space. To enhance model alignment and derive molecular representations with enriched semantics, we obtain local and global representations at various levels of the model, implementing contrastive learning through AMCLoss separately for these representations. Extensive experiments showcase that MolMVC attains state-of-the-art performance across various datasets and multiple tasks. In addition, the results from visualization experiments underscore the high performance and interpretability of our proposed molecular representation.

## 2 Materials and methods

### 2.1 Materials

For the pre-training dataset, we leverage the PCQM4Mv2 dataset sourced from the OGB Large-Scale Challenge (Hu *et al.* 2021), which has 3.4 million molecular data instances. For the downstream tasks, we select Molecular Property Prediction (MPP), Cancer Drug Response (CDR), Drug-Target Binding Affinity (DTA), and SARS-CoV-2 drug repositioning. For MPP, we adopt six classification benchmark datasets sourced from the widely used MoleculeNet (Wu *et al.* 2018). Following previous work (Xia *et al.* 2023), we use scaffold splitting (Ramsundar *et al.* 2019) to divide each dataset into 8:1:1 for training, validation, and testing, respectively. For other drug-related tasks, we use the same dataset and data segmentation as the competitive methods. More details about the datasets and input features can be found in Supplementary Materials.

### 2.2 Overview of MolMVC

**Figure 1.**
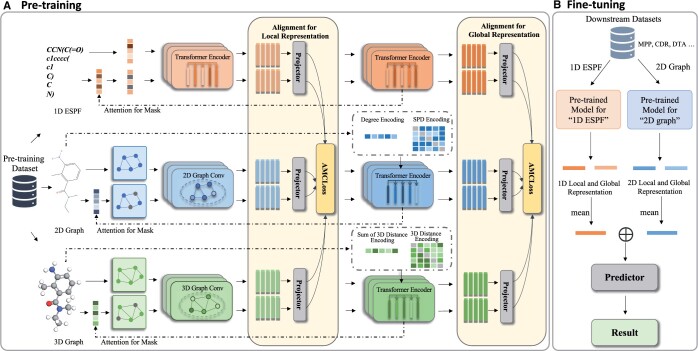
Flowchart of MolMVC framework including pre-training and transfer learning stages. (A) For the pre-training stage, MolMVC utilizes multi-view contrastive learning to mine the multi-perspectives information of three modalities of molecular data. (B) In the fine-tuning stage, due to the scarcity of 3D data for downstream tasks, MolMVC only uses 1D and 2D molecular representation enhanced by 3D molecular information to improve the performance of drug-related tasks. Further elaboration on these stages is expounded upon in Section 2.2.

As depicted in Fig. 1, our MolMVC framework comprises a pre-training stage and a transfer learning stage. In the pre-training stage, we commence by acquiring representations of the original samples through 1D, 2D, and 3D molecular encoders. Subsequently, we introduce a novel attention-guided augmented sample generation method, incorporating prior knowledge, to generate positive samples for different molecular data modalities. We then obtain the molecular representations for these augmented samples. Following this, we introduce the AMCLoss for contrastive learning, aligning the multi-view molecular representations concurrently. To capitalize on the hierarchical attributes of the encoders at both local and global levels, we perform contrastive learning for representations of the three molecular data modalities at different levels. Moving to the transfer learning stage, molecular 3D information for downstream tasks is scarce. Benefiting from our pre-training strategy, we exclusively utilize the 1D and 2D molecular encoders, enriched by 3D geometry information. To obtain a representation with multi-level information, we use a mean operation for both local and global presentations from the two molecular modality encoders. Subsequently, we concatenate these two representations and use them as inputs to the predictor for downstream tasks.

### 2.3 Backbone

Depending on the molecular data modality, we use specific encoders to embed latent information. Taking an arbitrary molecule *m* as an example, which comprises *d* atoms. For the 1D ESPF, we use the Transformer encoder, known for its efficacy in extracting sequence information through a multi-head attention mechanism. The Transformer, originally designed for Natural Language Processing tasks (Vaswani *et al.* 2017), proves to be a suitable choice for mining contextual information from ESPF sequences. To elaborate, ESPF successfully identifies crucial functional groups, enabling the division of a SMILES representation into *e* segments based on a substructure vocabulary *V*. This process yields an ESPF sequence, denoted as $E_{m}=\left\{S_{1},\ldots ,S_{e}\right\}$, where S∈V. Subsequently, this ESPF sequence is encoded into an embedding within a high-dimensional space using an embedding layer. To incorporate positional information for each substructure, a positional embedding is generated through one-hot encoding and another embedding layer. The combination of these two embeddings yields the input $H_{m,\mathrm{input}}^{1d}$ for the Transformer encoder:

(1) $$H_{m,\mathrm{input}}^{1d}=E_{m}+P_{m},$$

where *E_m_* is ESPF embedding and *P_m_* is positional embedding. A Transformer encoder consists of stack blocks. Each block contains a multi-head attention layer and a feed-forward layer. The calculation process of Transformer can be found in Supplementary Materials.

For the 2D and 3D graph data, in contrast to other SSL methods for molecular representation learning, we use a Graph Transformer. This Transformer is capable of simultaneously capturing short-range information from neighboring atoms and long-range information from the entire molecule. The Graph Transformer comprises two main components: a graph encoder and a Transformer encoder. In alignment with previous works (Liu *et al.* 2021a, Xia *et al.* 2023), we opt for the Graph Isomorphism Network (GIN) (Xu *et al.* 2018) for 2D topology graph data and SchNet (Schütt *et al.* 2017) for 3D geometry graph data. GIN, a variation of Graph Neural Network, is known for its quantifiable generalization ability. SchNet is a geometric representation learning method which can model pairwise interaction of atoms. The calculation process of GIN and SchNet can be found in Supplementary Materials.

After the processing of GIN and SchNet, we can generate the atomic representations $A_{m}^{2d}$ and $A_{m}^{3d}$ of *m* as the input of subsequent Transformer encoders. Different from the general Transformer, we use the similar encoding strategies with Ying *et al.* (2021) and Shi *et al.* (2022) to enhance positional encodings and self-attention layer with prior knowledge for 2D and 3D Graph Transformer respectively. For 2D Graph Transformer, we introduce degree of atoms for positional encoding to encode centrality information and shortest path distance (SPD) for self-attention layer to encode topological relationships between atom pairs:

(2) $$H_{m,\mathrm{input}}^{2d}=A_{m}^{2d}+D_{m},$$

 

(3) $$\begin{array}{c} {\;\text{head}}_{i}^{2d}=\begin{array}{c} \text{softmax}\left(\frac{Q_{n}^{2d}K_{n}^{2d}}{\sqrt{d}}+S_{m}\right)V_{n}^{2d}, \end{array} \end{array}$$

where *D_m_* is the degree embedding and *S_m_* is the SPD embedding. For 3D Graph Transformer, we introduce sum of 3D distance to encode spatial encoding and 3D distance to encode geometric relationships between atom pairs in a molecule, where the 3D distance refer to euclidean distance processed by the Gaussian Basis Kernel function:

(4) $$H_{m,\mathrm{input}}^{3d}=A_{m}^{3d}+E_{m}^{\mathrm{sum}},$$

 

(5) $$\begin{array}{c} {\;\text{head}}_{i}^{3d}=\begin{array}{c} \text{softmax}\left(\frac{Q_{n}^{3d}K_{n}^{3d}}{\sqrt{d}}+E_{m}\right)V_{n}^{3d}, \end{array} \end{array}$$

where $E_{m}^{\mathrm{sum}}$ is the embedding of the sum of 3D distance and *E_m_* is the 3D distance embedding.

### 2.4 Attention-guided augmentation with prior knowledge

One pivotal factor influencing the efficacy of contrastive learning lies in the quality of positive samples generated for the original samples. To ensure that our model captures crucial information from each molecular data modality effectively, we propose an innovative mask strategy for generating positive samples based on the attention scores of the Transformer and Graph Transformer. The attention score allows the model to assign varying levels of importance to input features through different weighted distributions. By leveraging the magnitude of attention scores, we can establish the importance ranking of input features in the model. Utilizing this ranking as guidance, we choose to mask a certain percentage (r%) of segments for 1D ESPF or atoms for 2D and 3D graphs with the highest attention scores. Here, *r* represents the masking ratio. We use a vector $m=(m_{1},m_{2},\ldots ,m_{d})$ to represent whether atoms are masked.

(6) $$m_{i}=\{\begin{array}{c} 1,\mathrm{rank}(i)\leq r\\ 0,\mathrm{rank}(i)>r \end{array}$$

where *rank*(*i*) is the attention score ranking of atom *i*. This strategy compels the model to learn critical information that has been intentionally masked, preventing the generation of overly similar positive pairs that could compromise the optimization objectives. Moreover, our augmentation strategy incorporates molecular prior knowledge. For 1D molecular data, ESPF serves as the target for data augmentation, providing substructure information. For 2D molecular data, we enrich the strategy by considering SPD and degree during the calculation of attention scores, while for 3D, we take into account 3D distance and the sum of distance. Consequently, we obtain augmented samples for each modality with distinct masks, enhancing the diversity of information in pre-training.

### 2.5 Adaptive multi-view contrastive loss and hierarchical contrastive learning

The objective of contrastive learning is to concurrently align positive samples and differentiate negative samples (Jaiswal *et al.* 2020). In the context of molecular contrastive learning, the selection of positive sample pairs can be broadly categorized into two strategies: constructing augmented samples and utilizing different modalities of molecular data. However, there remains a research gap in terms of effectively combining these two strategies. Drawing inspiration from the Supervised Contrastive Loss in computer vision (Khosla *et al.* 2020), which utilizes two positive sample sources–image augmentation and images within the same batch describing identical entities, we present an Adaptive Multi-View Contrastive Loss (AMCLoss). This loss is designed to treat the three modalities of molecular data and their corresponding augmented molecules as positive samples, utilizing their representations for contrastive learning. Given that positive sample pairs composed of samples from different views of molecules can take various forms, and the alignment difficulty differs for each type, we aim to balance the learning rate between different positive pairs during pre-training. To achieve this, we introduce the concept of the Dynamic Weight Average (DWA) (Liu *et al.* 2019b) scheme, originally developed for multitask learning. This scheme adjusts the weight of each task based on the rate of change of loss. For different types of molecular positive pairs, we calculate the loss ratio of adjacent epochs and use the softmax function to derive the weight of each positive pair. The specific calculation process of AMCLoss is as follows:

(7) $$\begin{array}{cc} {\text{AMCLoss}}^{p} & =\begin{array}{c} \sum_{i}\sum_{j}{\text{AMCLoss}}_{ij}^{p} \end{array}\\ & =\begin{array}{c} -\sum_{i}\sum_{j}\;\text{log}\;\frac{w_{ij}^{p}\;\text{exp}\;\left(z_{i}\cdot z_{j}\right)}{\sum_{o\in \mathrm{other}(M_{a})}\;\text{exp}\;\left(z_{i}\cdot z_{o}\right)}, \end{array} \end{array}$$

where *p* is the iteration index, *M_a_* is a set of index, which contains samples of all views of molecule *a*, $i\in M_{a},\;j\in M_{a}\backslash \{i\}$, *z* represents the molecular representation mapped by a nonlinear projection head, $\mathrm{other}(M_{a})$ includes the indexes of other samples unrelated to molecule *a* and $w_{ij}^{p}$ is the weight for positive pair *ij* in *p* iteration. The faster the learning speed of the positive pair, the lower its weight in the next iteration. And the weight for positive pair *ij* is calculated as follow:

(8) $$r_{ij}^{p-1}=\frac{{\text{AMCLoss}}_{ij}^{p-1}}{{\text{AMCLoss}}_{ij}^{p-2}},$$

 

(9) $$w_{ij}^{p}=\frac{K\;\text{exp}\;\left(r_{ij}^{p-1}\right)}{\sum_{j}\;\text{exp}\;\left(r_{ij}^{p-1}\right)},$$

where *K* is the number of types of molecular positive pair and it can ensure $\sum_{j}\left(w_{ij}^{p}\right)=K$.

To capture molecular information at both the local and global levels and facilitate the hierarchical alignment of the model, we introduce hierarchical contrastive learning based on the hierarchical characteristics of the backbones in MolMVC. For the Transformer designed to process 1D molecular sequence information, the lower layers predominantly handle lower-order semantic information, while the higher layers focus on higher-order syntactic information. In the case of the Graph Transformer utilized for processing 2D and 3D molecular graph information, the graph encoder primarily concentrates on local neighbor-level information, while the Transformer encoder addresses global molecular-level information. Specifically, we consider the output of the middle layer of the Transformer and the output of the graph encoder of the Graph Transformer as local-level representations, with their final output representing the global-level representation. By implementing contrastive learning at both levels, we achieve hierarchical contrastive learning, enabling the model to discern and align molecular features at different scales of abstraction.

## 3 Results

To assess the performance of MolMVC empirically, we first pre-train our model on the PCQM4Mv2 pre-training dataset. Following the pre-training phase, we conduct comprehensive downstream experiments, covering MPP, DTA prediction, CDR prediction, SARS-CoV-2 drug repositioning, and additional interpretability experiments. Given the limited availability of 3D molecular data, we only use 1D and 2D parts of the pre-trained model for downstream tasks. Detailed information on the experimental settings can be found in Supplementary Table S2 in Supplementary Materials.

### 3.1 MolMVC for molecular property prediction

For MPP, we compare our MolMVC with competitive SSL baselines that only focuses on molecular information, including EdgePred (Hamilton *et al.* 2017), AttrMask (Hu *et al.* 2019), GraphCL, GPT-GNN (Hu *et al.* 2020), JOAO (You *et al.* 2021), GraphLoG (Xu *et al.* 2021), 3D InfoMax, GraphMAE (Hou *et al.* 2022), GraphMVP, Mole-BERT. The evaluation metric is the area under the receiver operating characteristic curve (AUC). The performance comparison of MolMVC on molecular property prediction is presented in Table 1. We can draw the following conclusions from the results. First, MolMVC achieves the best performance on all the six benchmark datasets, demonstrating the effectiveness of our pre-training framework. Furthermore, MolMVC outperforms the current state-of-the-art method, Mole-BERT, by 6.1% in overall performance under the same experimental protocols, indicating significant improvements. Second, MolMVC performs well on datasets that encompass multiple tasks, such as ClinTox, Toxcast, and Sider. Notably, MolMVC achieves a performance improvement of 19.5% on the ClinTox dataset with two tasks and 6.6% on the ToxCast dataset with 617 tasks. We believe this is because our pre-training strategy can fully consider the views of molecules, allowing the model to fit the distribution of these datasets well and achieve significant improvement. Third, in terms of properties related to drugs, MolMVC excels, including BBBP, ClinTox, HIV and Sider. The robust performance demonstrates that MolMVC can generate informative molecular representations, effectively capturing the knowledge distribution of molecules, especially drug-related knowledge.

**Table 1.** Results of various methods for MPP with scaffold splitting.^a^

Methods	BBBP	BACE	ClinTox	HIV	Sider	ToxCast
EdgePred	67.3 ± 2.4	77.3 ± 3.5	64.1 ± 3.7	75.1 ± 1.2	60.4 ± 0.7	64.1 ± 0.6
AttrMask	65.2 ± 1.4	77.8 ± 1.8	73.5 ± 4.3	75.3 ± 1.5	60.5 ± 0.9	63.3 ± 0.6
GraphCL	67.8 ± 2.4	74.6 ± 2.1	77.5 ± 3.8	75.1 ± 0.7	59.8 ± 1.3	63.0 ± 0.4
GPT-GNN	64.5 ± 1.4	77.9 ± 3.2	58.3 ± 5.2	65.2 ± 2.1	58.1 ± 0.3	62.5 ± 0.4
JOAO	66.4 ± 1.0	73.2 ± 1.6	66.6 ± 3.1	76.6 ± 1.7	60.4 ± 1.5	62.8 ± 0.7
GraphLoG	68.7 ± 1.6	78.6 ± 1.0	75.7 ± 2.4	76.1 ± 0.8	59.6 ± 1.9	63.4 ± 0.6
3D InfoMax	69.1 ± 1.2	78.6 ± 1.9	62.7 ± 3.3	76.1 ± 1.3	56.8 ± 2.1	63.5 ± 0.8
GraphMAE	71.2 ± 1.0	78.2 ± 1.5	76.5 ± 3.0	76.8 ± 0.6	60.5 ± 1.2	63.6 ± 0.3
GraphMVP	70.8 ± 0.5	79.3 ± 1.5	79.1 ± 2.8	76.8 ± 0.6	60.2 ± 1.1	63.1 ± 0.2
Mole-BERT	71.9 ± 1.6	80.0 ± 1.4	78.9 ± 3.0	78.2 ± 0.8	62.8 ± 1.1	64.3 ± 0.2
MolMVC	**74.4 ± 0.2**	**85.9 ± 0.1**	**98.4 ± 0.1**	**78.7 ± 0.6**	**64.3 ± 0.7**	**70.9 ± 0.4**

a The results of baseline methods are taken from Xia *et al.* (2023). The best performance for each metric is marked in bold.

### 3.2 MolMVC for other drug-related tasks

To validate the utility of our molecular representations in drug-related tasks, we leverage the representations generated by pre-trained MolMVC for DTA and CDR tasks. We build two MolMVC* models by replacing the drug processing component of state-of-the-art methods in these tasks with a 2-layer deep neural network (DNN) that maps the molecular representations to an appropriate latent space. Specifically, we substitute GraphDTA for DTA prediction and DeepTTA for CDR prediction. The MolMVC* models lack any drug information except for MolMVC representations. For DTA tasks, the comparison methods include GraphDTA (Nguyen *et al.* 2021), WideDTA (Öztürk *et al.* 2019), DeepDTA (Öztürk *et al.* 2018), SimBoost (He *et al.* 2017), and KronRLS (Cichonska *et al.* 2018). The metrics are Mean Square Error (MSE) and Concordance Index (CI). In CDR tasks, the comparison methods are DeepTTA (Jiang *et al.* 2022), DeepCDR (Liu *et al.* 2020), tCNNs (Liu *et al.* 2019a), CDRscan (Chang *et al.* 2018), and MOLI (Sharifi-Noghabi *et al.* 2019). The evaluation metrics are Pearson’s correlation coefficient (PCC) and Spearman’s correlation coefficient (SCC). As indicated in Tables 2 and 3, MolMVC* models achieve state-of-the-art performance across all datasets and metrics. This demonstrates that our representations possess strong generalization ability and effectively contribute to drug-related tasks. In addition, we conduct a runtime analysis of using our representations in downstream tasks on the same server. For the DTA task, the training cost of MolMVC* model and GraphDTA is 5.0 h and 5.4 h on the Davis dataset, while 80.4 h and 88.3 h on the Kiba dataset. For the CDR task, the training cost of MolMVC* model and DeepTTA is 0.3 h and 0.8 h. The results indicate that our representations not only enhance performance in drug-related tasks but also significantly reduce learning costs.

**Table 2.** Results of various methods for DTA.^a^^,^^b^

Methods	Davis			Kiba		
	CI	MSE	Cost	CI	MSE	Cost
KronRLS	0.871	0.379		0.782	0.411	
SimBoost	0.872	0.282		0.836	0.222	
DeepDTA	0.878	0.261		0.863	0.194	
WideDTA	0.886	0.262		0.875	0.179	
GraphDTA	0.893	0.229	5.4h	0.882	0.147	88.3h
${\text{MolMVC}}^{*}$	**0.894**	**0.225**	**5.0h**	**0.892**	**0.146**	**80.4h**

a The results of baseline methods are taken from Nguyen *et al.* (2021). The best performance for each metric is marked in bold.

b ${\text{MolMVC}}^{*}$ is a variant which uses a simple DNN to handle MolMVC representation to replace GraphDTA’s drug processing component.

**Table 3.** Results of various methods for CDR.^a^

Methods	PCC	SCC	Cost
MOLI	81.3	78.2	
CDRscan	87.1	85.2	
tCNNs	91.0	88.9	
DeepCDR	92.3	89.8	
DeepTTA	94.1	91.4	0.8h
${\text{MolMVC}}^{*}$	**94.3**	**93.7**	**0.3h**

a The results of baseline methods are taken from Jiang *et al.* (2022). The best performance for each metric is marked in bold.

Furthermore, we explore the application of MolMVC in drug repositioning and prove MolMVC has the ability to search for potential antiviral COVID-19 drugs. The results are shown in Supplementary Fig. S1 and Supplementary Table S3 in Supplementary Materials Fig. S2.

### 3.3 Ablation study

To examine the essential components of MolMVC, we design five variants of MolMVC. **NoPretrain** removes pre-training scheme. **NoLocal** only conducts contrastive learning at global level. **No3D** pre-trains the model without using 3D molecular data. **RandomMask** adopts a augmentation strategy of randomly masking. **SupLossPretrain** uses the same type of loss SupLoss (Khosla *et al.* 2020) as AMCLoss, which is state-of-the-art. We perform ablation studies on the classic BBBP, BACE, and Sider datasets in MPP task. The results are depicted in Fig. 3. The noticeable decrease in performance for **NoPretrain** variant underscores the effectiveness of our pre-training scheme in learning potential molecular knowledge without labeled data. The experiment involving **NoLocal** demonstrates that hierarchical contrastive learning empowers MolMVC to comprehensively capture information from different levels of molecules. When pre-training the model without 3D molecular data (**No3d**), the consistently inferior performance compared to MolMVC indicates that introducing 3D data during pre-training effectively infuses molecular geometric knowledge into 1D and 2D encoders, thereby enhancing performance even in the absence of 3D information. The performance of **RandomMask** illustrates that our attention-guided augmentation with prior knowledge generates higher-quality augmented samples. The experiment on **SupLossPretrain** shows our AMCLoss can help achieve better pre-training effects by balancing the learning rate of different types of positive pairs.

**Figure 2.**
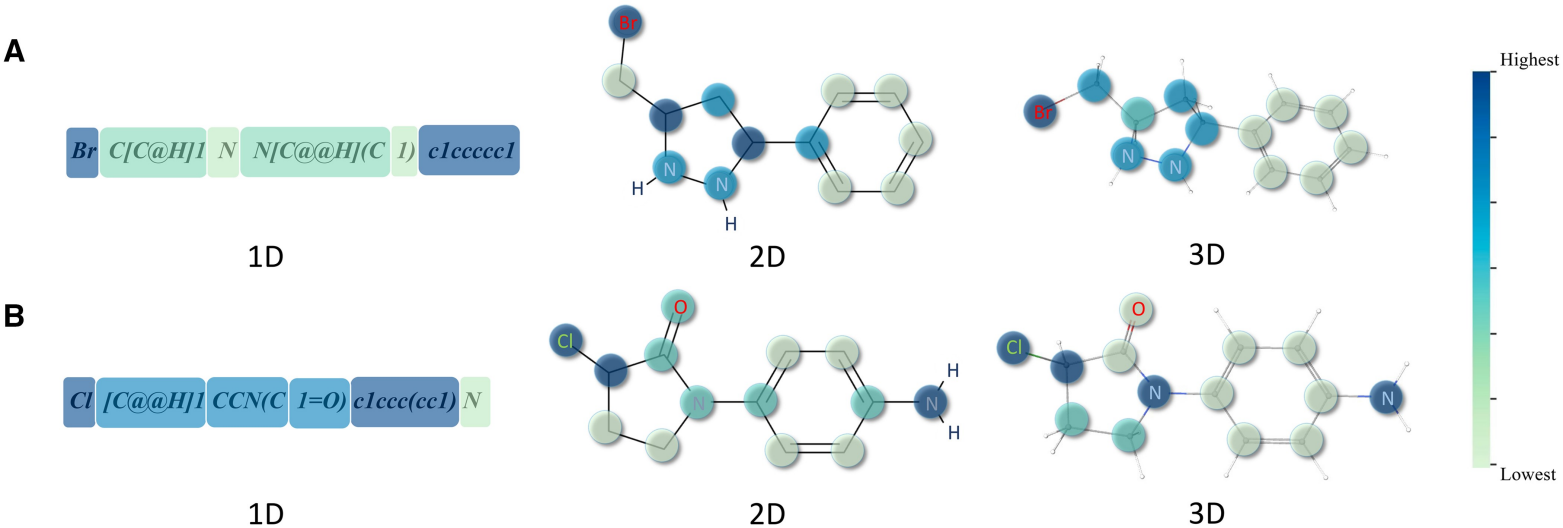
Visualization of three modalities of molecules based on attention weight coloring. (A) and (B) are two randomly selected examples from pre-training dataset.

**Figure 3.**
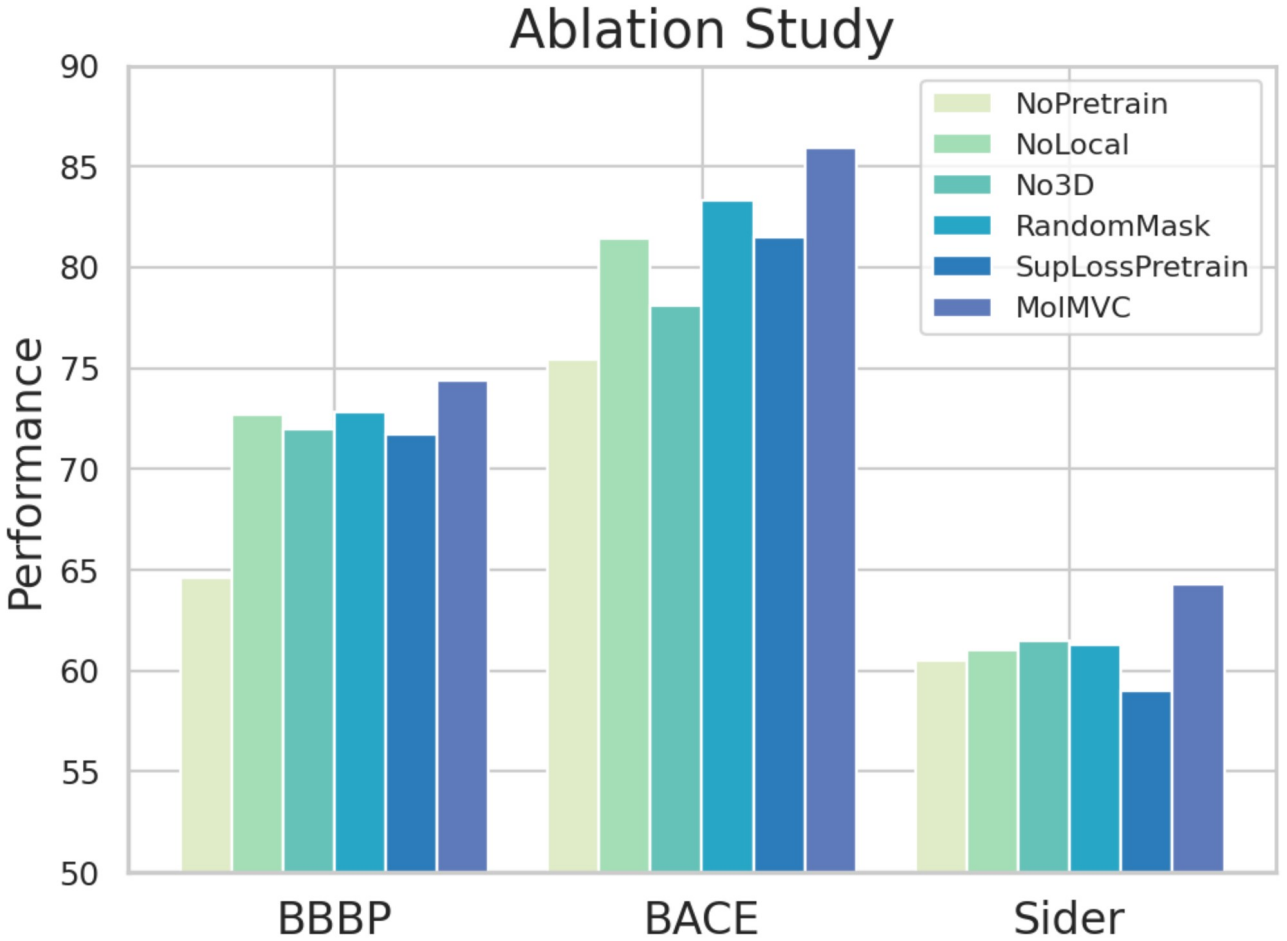
Results of ablation study on classic BBBP, BACE and Sider datasets.

To further explore the role of our pre-training process, we study the distribution and alignment of molecular representations before and after pre-training. The results demonstrate that our pre-training scheme aligns different modalities and ensures the learned molecular representation distribution is coherent and reasonable. The results are provided in Supplementary Figs S2 and S3 in Supplementary Materials.

### 3.4 Attention scores unveil crucial molecular substructures

**Figure 4.**
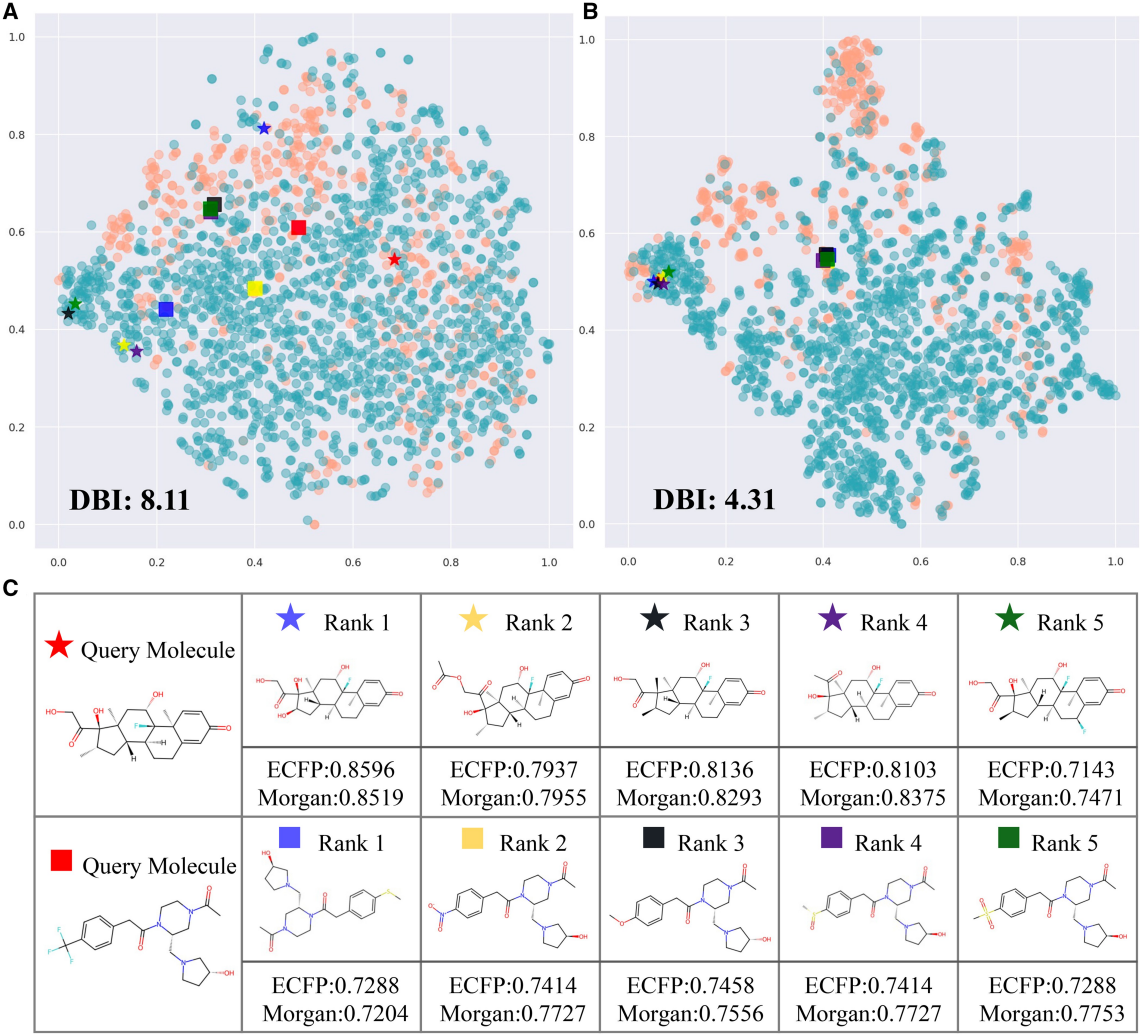
Investigation of representation. (A) and (B) are t-SNE visualization of representation of BBBP dataset with and without pre-training. (C) is the results of molecular retrieval.

To investigate whether our attention scores can recognize key parts in molecules, we visualize the attention scores for the three data modalities of molecules. As shown in Fig. 4, we randomly select two molecules from the pre-training dataset as examples. The color of each section or atom corresponds to a color bar gradient, with the lightest at the bottom and the darkest at the top, varying by proportion. From the visualized results, we have the following observations. Firstly, the attention scores of the three data modalities all focus on halogen atoms: chlorine in Fig. 4A and bromine in Fig. 4B. Halogens are commonly used to enhance the lipid solubility of molecules, aiding drugs in passing through biofilms (Jaiswal *et al.* 2020). Secondly, in 2D and 3D, nitrogen-containing groups have higher attention scores. Nitrogen-containing groups typically play crucial roles in living organisms, participating in the formation of hydrogen bonds, interacting with biomolecules such as proteins and nucleic acids, or serving as targets for drug molecules (Vinogradov 1979). Thirdly, the 2D attention score focuses more on the connections between different substructures. Lastly, the encoders of the three modalities exhibit different attentional patterns, resulting in the masking of different parts of the molecules. This diversity increases the information contained in positive sample pairs during the pre-training stage.

### 3.5 Investigation of molecular representation of MolMVC

To further assess the quality of our representations, we use t-SNE (Van der Maaten and Hinton 2008) to dimensionality reduce and visualize the representations of the BBBP dataset generated from MolMVC with and without pre-training. In Fig. 4A and B, the blue and orange dots represent positive and negative samples, respectively. It is evident that even without any knowledge about the BBBP dataset, after pre-training, dots of the same class cluster together, while those of different classes disperse. A lower Davies Bouldin Index (DBI) supports that the representations from pre-trained MolMVC achieve better clustering performance.

We also conduct molecular retrieval experiments. Two molecules are randomly selected as query molecules in the BBBP dataset, and the remaining molecules are reference molecules. The cosine similarity of MolMVC representations between the query molecules and reference molecules is calculated, and the top five molecules are shown in Fig. 4C. Notably, these molecules exhibit similar structures. Conventional fingerprints, Morgan and ECFP, are used to verify their similarity. The results indicate high similarity for these molecules. In addition, Fig. 4A and B shows these query and reference molecules. After pre-training, molecules that were initially distant become closer, while molecules that were initially close still remain close. More results are shown in Supplementary Fig. S4 in Supplementary Materials. These results demonstrate that our pre-training scheme aids the model in learning molecular knowledge and uncovering intrinsic connections between molecules.

## 4 Conclusion

In this paper, we introduce MolMVC, a novel multi-view contrastive learning framework for molecular representation, and provide our pre-trained model to enhance drug-related tasks. MolMVC simultaneously consider molecular 1D, 2D, and 3D modalities data, utilizing the proposed AMCLoss to combine multi-view information in contrastive learning. Our attention-guided augmentation strategy with prior knowledge ensures the generation of high-quality positive samples for each molecular data modality. Extensive experiments showcase MolMVC’s state-of-the-art performance in multiple benchmarks, demonstrating its effectiveness in drug-related tasks. In addition, MolMVC exhibits strong capabilities in COVID-19 drug repositioning. Interpretability experiments affirm that our representation encapsulates comprehensive molecular knowledge.

## Supplementary Material

btae386_Supplementary_Data

## Data Availability

The data underlying this article are available in https://github.com/Hhhzj-7/MolMVC.
